# SAW Sensors for Chemical Vapors and Gases

**DOI:** 10.3390/s17040801

**Published:** 2017-04-08

**Authors:** Jagannath Devkota, Paul R. Ohodnicki, David W. Greve

**Affiliations:** 1 National Energy Technology Laboratory, Pittsburgh, PA 15236, USA; dg07@andrew.cmu.edu; 2 Department of Materials Science and Engineering, Carnegie Mellon University, Pittsburgh, PA 15213, USA; 3 Department of Electrical and Computer Engineering, Carnegie Mellon University, Pittsburgh, PA 15213, USA

**Keywords:** acoustic waves, acoustoelectric effect, interdigital transducer, mass loading, piezoelectric effect, radiofrequency, sensing layer, viscoelasticity

## Abstract

Surface acoustic wave (SAW) technology provides a sensitive platform for sensing chemicals in gaseous and fluidic states with the inherent advantages of passive and wireless operation. In this review, we provide a general overview on the fundamental aspects and some major advances of Rayleigh wave-based SAW sensors in sensing chemicals in a gaseous phase. In particular, we review the progress in general understanding of the SAW chemical sensing mechanism, optimization of the sensor characteristics, and the development of the sensors operational at different conditions. Based on previous publications, we suggest some appropriate sensing approaches for particular applications and identify new opportunities and needs for additional research in this area moving into the future.

## 1. Introduction

Wohltjen and Dessy introduced SAW technology for chemical sensing applications in 1979 [1,2]. Since then, chemical sensors based on this technology have continuously received research and technological attention. Important advantages of SAW sensors include ultra-high sensitivity, excellent response time, small size, low cost, ability to work in wired and wireless mode, compatibility with modern fabrication technologies, and planar structure [3,4,5,6]. In addition, these sensors offer excellent selectivity, fast response, reversibility, linearity, and stability upon proper selection of sensing layers, piezoelectric substrates, and interdigital transducers (IDTs) [7,8]. These sensors are expected to fulfil the increasing demand of high performance chemical sensors in industries, military, pollution and emissions control, combustion exhausts, and other disciplines for detecting and monitoring various inorganic gases, volatile organic chemicals (VOCs), and chemical warfare agents (CWAs) amongst others.

All types of SAW sensors rely on changes in the propagation characteristics of SAWs due to an interaction between the waves and surface layers or the nearby environment [9,10,11,12]. These waves have a penetration depth of the order of the wavelength so that most of the energy density is confined to the near-surface region. This makes the waves and hence the devices based on them highly sensitive to any physical or chemical changes on or near the surface. Furthermore, the operational frequency of SAW devices can be set in a wide range (MHz–GHz) which helps tuning the sensitivity and opens the possibility of operating these devices in wireless mode. This platform has already been used for sensing several physical quantities (e.g., temperature, pressure, and stress), many chemicals, and biological entities [4,13,14,15].

For chemical and biological sensing, SAW devices are coated with a layer of suitable material such as polymers, metals, and metal oxides [16,17]. Any change in the mass, mechanical, or electric properties of this layer upon exposure to the foreign molecules perturbs the surface waves enabling the devices to use as sensors [18,19]. Numerous review reports summarizing the progress in the chemical sensing application of Rayleigh wave-based SAW devices can be found in the literature [4,8,13,16,17,19,20,21,22,23]. The sensors based on other surface waves such as Love and shear horizontal SAW (SH-SAW) waves have also been developed for detection of various chemicals and biological entities in fluidic media. Readers interested in the applications of the SAW sensors in liquid media are directed to [14,21,24,25,26]. In this review, we give a brief introduction to the topic of sensors based on Rayleigh waves and discuss their advances in sensing chemicals in the gaseous phase.

## 2. Fundamental Concepts

The central idea of surface acoustic devices involves the interconversion of electric and mechanical energies using the piezoelectric effect [27]. The operation of these devices consists of three major aspects: (i) excitation of the acoustic waves in a piezoelectric material; (ii) modulation of the wave characteristics in the propagation path; and (iii) successful detection. The excitation and detection of the surface elastic waves can be achieved using IDTs, whereas the propagation characteristics are modulated in their path due to interactions with a number of physical or chemical parameters. In case of chemical sensing applications, these changes are induced via the mass, viscoelasticity, and conductivity of a recognition layer coated in their path upon exposure to foreign chemicals [2,28,29,30]. These changes can be experimentally detected in terms of frequency, phase, and insertion loss of corresponding electric signal and correlated to the quantity that caused the change [1,9,31,32,33]. Below, we give an overview of the fundamentals of SAW chemical sensors.

### 2.1. Rayleigh Waves

Rayleigh waves are a type of surface elastic waves, which are a member of broad acoustics wave family, and are similar to the mechanical waves on the Earth surface during an earthquake [30,34]. These waves propagate along the surface of a material and decay exponentially into the depth of the material (penetration depth ~ 1 wavelength) so that most of their energy density is localized in the near surface region. They propagate with a velocity slightly less than that of transverse waves in the material (and ~10^−5^ times the velocity of electromagnetic waves). The particles of their propagating medium are displaced in longitudinal and shear vertical directions so that the wave propagation follows a retrograde elliptical path [30]. These waves were first described in isotropic solids by Lord Rayleigh in 1885 [34,35]. In 1955, Stoneley gave a detailed analysis of their propagation in anisotropic media [36]. However, surface waves began to be applied in electronic devices only after White and Voltmer [37] demonstrated in 1959 that these waves could be easily generated in piezoelectric materials using IDTs [34].

There are several other variants of surface waves such as SH-SAW, Love, Stoneley, Lamb, and Leaky waves [30]. In SH-SAW, the particles of the propagating medium are displaced horizontally rather than vertically. Love and Stoneley waves are observed in layered structures and propagate with velocities different from that of Rayleigh waves. All these surface waves are distinct from the bulk acoustic waves (BAWs) which propagate across the body of a material [38,39]. The propagation characteristics of different modes of the acoustic waves are well known and their applications have been realized in many disciplines [14,39]. Among them, sensors based on the Rayleigh mode are found to be extremely sensitive to a number of quantities. In fact, a variety of sensors to detect and monitor different quantities such as temperature, pressure, humidity, chemical vapors, and gases have been developed using these waves. However, these waves are not typically suitable for use in liquid media due to a large loss of acoustic energy caused by a coupling to longitudinal waves in the liquid, as observed by Calabrese et al. [25,26]. Alternatively, sensors based on other surface waves such as SH-SAW and Love waves have been successfully employed in fluidic media [21,24].

### 2.2. Piezoelectric Materials

In 1880s, Pierre and Paul-Jacques Curie showed that some anisotropic materials can generate an electric polarization under an external mechanical strain (piezoelectric effect) or a mechanical strain under an external electric field (reverse effect) [40,41]. The efficiency of this electromechanical conversion in a material is measured by its electromechanical coupling coefficient, *K*^2^ = *e*^2^/*c*ε, where *e*, *c*, and ε are the piezoelectric coupling, stiffness, and the dielectric coefficient, respectively of the material for a given propagation direction.

The materials with large *K*^2^ and zero temperature coefficient of frequency (TCF) are ideal materials for developing SAW chemical sensors. A low value of *K*^2^ causes a large attenuation of the waves and a poor conversion of electric-acoustic energy. Similarly, a strong temperature dependence may cause variation in the center frequency not associated with chemical changes. The substrate material as well as its crystal cut and orientation (i.e., axis rotation) affect these and other parameters such as polarization (Rayleigh, SH-SAW, Lamb, Love etc. modes), velocity, and power flow angle of the excited waves [24,42,43,44,45]. For example, Rayleigh waves are excited in Y-Z LiNbO_3_ (where Y-Z stands for Y-cut, Z-propagating) and 128° Y-X LiNbO_3_ substrates whereas SH-SAW are shown to excite and propagate in 64° Y-X LiNbO_3_ and 36° Y-X LiTaO_3_ [24,30,46]. Similarly, only the Rayleigh mode is susceptible to propagate as a surface wave in a homogeneous semi-infinite medium [47]. On the other hand, even though Rayleigh waves can be excited in both Y-Z LiNbO_3_ and 128° Y-X LiNbO_3_ substrates, the waves propagate with different velocities (~3488 m/s, and ~3992 m/s, respectively) in these substrates [43]. Another important characteristic of piezoelectric materials is their Curie and/or phase transition temperature (T_max_) that sets an upper limit for the operating temperature. While the Curie temperature represents an upper limit for piezoelectric behavior, often the operation is limited to much lower temperatures due to chemical decomposition, increased attenuation, phase transition, increased structural disorder, decreased mechanical quality factor, low electrical resistivity, and loss of oxygen to the environment at higher temperatures [48].

Among the most commonly explored piezoelectric substrates for generation of Rayleigh waves are ST-X quartz and Y-Z LiNbO_3_. The former has nearly zero TCF whereas the latter has a much larger value of *K*^2^. Other materials such as langasite (La_3_Ga_5_SiO_14_) [44] and the films of zinc oxide (ZnO) [47], aluminum nitride (AlN) [49], and polyvinylidene fluoride (PVDF) [50] have also been used as the substrate materials. In addition, there have been some efforts of using layered piezoelectric substrates (ZnO/Diamond/Si, SiO_2_/ST-90X Quartz etc.) for generating SAW waves of different modes (e.g., Love waves, Bluestein-Gulyaev waves) [49,51]. Studies have shown that the mode of excited SAWs, their phase velocity, and the value of *K*^2^ can vary with the physical parameters such as the thickness of the piezoelectric films [8,49,51]. Table 1 lists some popular piezoelectric substrates and their characteristics as collected from the literature [30,42,43,44,45,47,48,52,53,54,55,56].

### 2.3. Interdigital Transducers

**Figure 1:**
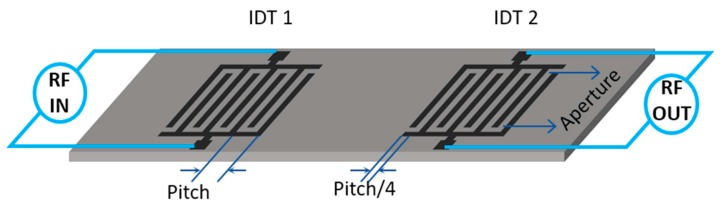
Schematic of a SAW delay line. Shown are the input and output IDTs on a piezoelectric substrate.

IDTs are integral parts of the SAW devices. They are periodic metallic electrodes (fingers) deposited on a substrate in the form of two combs intercrossing from opposite sides. Their major function in SAW devices is to convert the electrical radio frequency (RF) signal to SAWs on piezoelectric surfaces and vice versa. There can be several types of IDT designs depending upon the applications. Figure 1 shows two sets of a simple type of IDTs on a piezoelectric substrate. In their simplest form, all fingers are identical, separated by a spacing equal to their width, and every alternate finger is from opposite bus-bar. When a time varying electric signal is applied to an IDT (input IDT), the alternate polarity of the electrodes causes a periodic compression and rarefaction of the piezoelectric material thereby generating an acoustic wave. The excited acoustic waves travel along the material’s surface on both sides of the IDT. Associated with the propagating wave is an electric field (in case of piezoelectric substrates) that protrudes from the surface and interacts with any overlayers present. Finally, these waves are converted back into an electric signal when they are incident on the second IDT (output IDT).

The design, geometry, number of fingers, delay path length, and material properties of the IDTs significantly affect the characteristics of SAW waves such as the operational frequency, bandwidth, amplitude attenuation, divergence etc. For instance, there can be some IDT designs that emit the acoustic waves in a preferred direction rather than emitting in both sides homogeneously [57]. The IDTs can emit SAWs most efficiently when the wavelength Λ of the waves matches the pitch of the IDT fingers (center to center distance between the two consecutive fingers in a comb). Also, the waves propagate approximately perpendicular to the fingers of the IDT and divergence decreases with increasing finger length. The number of fingers, on the other hand, affects the fractional bandwidth of the acoustic waves. The electrical impedance of the IDT is also important for efficient coupling of electrical energy. Details on the effect of IDT properties on SAW wave characteristics can be found in the literature [8,58].

The IDTs are fabricated using stable materials with high electrical conductivity and good mechanical adhesion with the piezoelectric substrate. In principle, any metal can be used to fabricate IDT in SAW devices, but material selection becomes critical when application is sought at high temperature or in a harsh environment. At high temperatures, many materials become unstable due to low melting points or chemical reactions with the substrate and environment [29] or can degrade due to agglomeration, recrystallization, and dewetting effects [48] thereby limiting their use as IDTs. Some widely-used IDT materials include aluminum, platinum, platinum-based alloys (e.g., Pt-Ir, Pt-Rh, Pt-Zr, Pt-Rh/ZrO_2_), copper, gold, tungsten, titanium, and conducting ceramics (e.g., La_0.65_Sr_0.35_MnO_3_, La_0.6_Sr_0.4_Co_0.2_Fe_0.8_O_3_). Experimentally, these materials can be deposited on the piezoelectric substrates by any metal deposition technique followed by photolithography to pattern into a desired IDT design [14].

### 2.4. Working Principle

**Figure 2:**
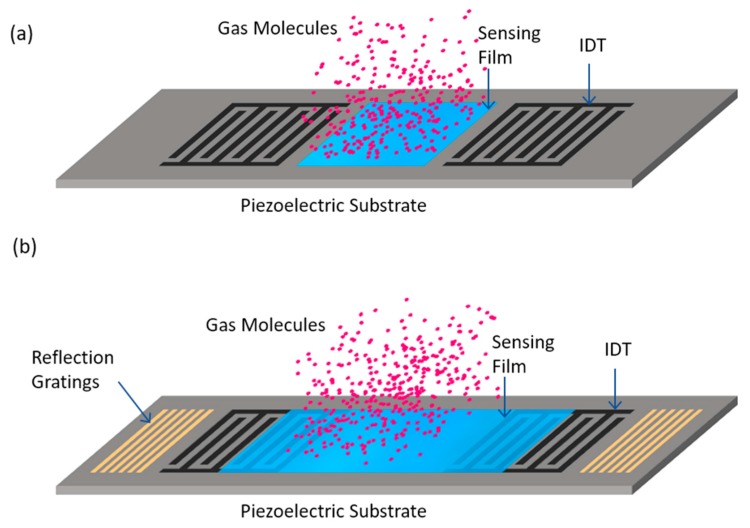
Schematics of SAW chemical sensors: a two-port delay line (**a**) and a resonator (**b**) with sensing overlayers and target analyte vapors.

SAW sensors are indirect probes of various physical and chemical quantities [4,27]. The presence of an entity in the propagation path of the surface waves causes a change in the phase velocity and amplitude of the waves. For chemical sensing, these changes are induced by variation in properties (to be discussed below) of a coated sensing layer on the piezoelectric transducer upon exposure to target analytes (Figure 2). By detecting these changes at the output IDT via the converted electric signal, one can obtain quantitative information about the analyte [2,3,4,13].

To excite the SAWs and detect the changes, two types of device configurations, namely the delay lines and the resonators (Figure 2a,b, respectively), are commonly used [6]. A typical ‘delay line’ SAW sensor consists of two IDTs deposited on a piezoelectric substrate at a certain separation, one for input and one for output of the electric signal. The region between the IDTs is coated with a recognition layer for interaction with foreign chemical vapors or gases. This region creates a delay in time between the input and output signals based on its length and the SAW velocity. Figure 2a shows a two-port delay line configuration of the SAW devices. It is also possible to use a single IDT for both purposes, the excitation and detection, by providing a reflector (usually another IDT). This is the one port or reflective delay line configuration. A delay line requires sufficient impedance matching for a tolerable insertion attenuation [59]. In addition, oscillator circuit design becomes complicated in this configuration as it offers relatively large phase changes (~2π) in its passband. However, this configuration is simple and practical for sensing applications.

The second configuration type of the devices is the ‘resonator’ that consists of two IDTs for emission and detection of the acoustic waves and grating reflectors are placed outside of each IDT so that a resonating cavity is formed in between. This structure is called the two-port resonator (Figure 2b). If a single IDT is used for the input and output signals, the configuration is called the one-port resonator. There can also be some modifications in these configurations [4,8]. In resonators, sensing layer can be deposited on to the IDTs and the impedance matching requirement is not as stringent because they have relatively smaller insertion attenuation. Also, the oscillation design is simple as this configuration offers relatively small phase change (~π) in the passband [6,59].

Both of these configurations have the same mechanism response and similar output characteristics [6]. Either of these configurations can be used to measure the changes in the phase velocity (v) and attenuation (α) of the acoustic waves upon exposure to gases. As the attenuation can suffer from undesired electromagnetic interferences, SAW sensor response is usually obtained in terms of velocity change to avoid such interferences [5,31]. Experimentally, the velocity change is evaluated by measuring the shift in center (resonance) frequency (f) or phase (ϕ) of the wave [9]. The measured changes in the center frequency and the phase with and without gas exposure are related to the phase velocity as [9]:

(1) $$\frac{\Delta v}{v_{0}}=-\frac{\Delta \phi }{\phi_{0}}=\frac{\Delta f}{f_{0}}$$

where$\;v_{0}$, $\phi_{0}$, and$\;f_{0}$ are the output velocity, phase, and center frequency of the SAW wave in unperturbed state whereas v, ϕ, and f are those in perturbed states.

In case of delay lines, it is necessary to measure small changes in the delay time. This can be accomplished by direct measurements of the pulse delay. However, the velocity changes are usually small enough that measurement of center frequency or phase changes is more practical. The phase difference can be determined by using quadrature detection [60,61]. On the other hand, a resonator configuration requires measuring its resonance frequency as the sensor response and relate it to the velocity change using Equation (1) [62,63]. Different types of measurement procedures of SAW sensors can be found in the literature [1,5,60,61,64,65,66,67].

### 2.5. Interacting Factors

The measured change in SAW velocity and attenuation is the sum of the influences of all possible internal and external factors to the propagating waves. In general, the factors that may interact with the acoustic waves can be categorized as the mass-loading (m), mechanical ($p_{mech}$) factors (viscosity, elasticity), electric ($p_{ele}$) factors (conductivity, permittivity etc.), and environmental ($p_{env}$) factors (temperature, pressure, humidity etc.). A perturbation-based investigation has been made to evaluate the effect of these factors quantitatively and shown that the net change in the velocity and attenuation is the sum of the perturbations caused by each [52,68]. The net perturbation on the wave propagation characteristics can be expressed as [33,52,69,70]:

(2) $$\frac{\Delta \gamma }{k_{0}}=\frac{\Delta \alpha }{k_{0}}-j\frac{\Delta v}{v_{0}}=\frac{\partial \gamma }{\partial m}\Delta m+\frac{\partial \gamma }{\partial p_{mech}}\Delta p_{mech}+\frac{\partial \gamma }{\partial p_{ele}}\Delta p_{ele}+\frac{\partial \gamma }{\partial p_{env}}\Delta p_{env}$$

where $\gamma \;(=\alpha +jk;\;j=\sqrt{-1}$ is the complex propagation coefficient of the propagating wave and $k_{0}$ (k=2π/Λ is the wave number associated with the wavelength Λ) is the wave number in unperturbed state.

In SAW chemical sensors (Figure 2), the propagating surface waves can interact with the overlayer in three distinct ways:

(i) Variation in propagation velocity caused by the mass of the overlayer: Absorption of molecular species changes the mass of the overlayer and thus the velocity of the surface wave [30,57,71,72,73,74,75].
(ii) Changes in propagation velocity or attenuation by the acoustoelectric effect: The surface wave is associated with an electric field protruding from the surface. Overlayers alter the stored energy in the electric field. The change in stored energy changes the electric properties (conductivity, permittivity etc.) and hence the propagation velocity. The wave is attenuated if the overlayer is of finite conductivity (i.e., resistive films) causing a net dissipation of energy [52,76,77,78,79].
(iii) Variation in propagation velocity by viscoelasticity: The viscoelastic properties (elasticity, viscosity) of the ovarlayer can be influenced by absorption of molecular species thereby causing a strain in it. This strain is partly transferred to the substrate and thus affects the wave propagation [80,81,82,83,84,85].

For an acoustically thin (thickness h<<Λ), non-conducting, non-piezoelectric, and isotropic layer, Wohltjen et al. [30] in 1984 expressed an equation for relative change in velocity due to changes in mass and stiffness of the layer referring to a perturbation analysis by Auld [68]. In 1985, Ricco et al. [52] derived an equation for velocity and attenuation change caused by a conducting otherwise similar film. The net changes due to the interactions for an acoustically thin, non-piezoelectric, and isotropic film can be written as [30,52,68]:

(3) $$\frac{\Delta v}{v_{0}}=-C_{m}f_{0}h\Delta \rho +C_{e}f_{0}h\Delta \left[\frac{4\mu }{v_{0}^{2}}\left(\frac{\lambda +\mu }{\lambda +2\mu }\right)\right]-\frac{K^{2}}{2}\Delta \left[\frac{\sigma_{s}^{2}}{\sigma_{s}^{2}+v_{0}^{2}C_{s}^{2}}\right]$$

and:

(4) $$\frac{\Delta \alpha }{k}=-\frac{K^{2}}{2}\Delta \left[\frac{v_{0}C_{s}\sigma_{s}}{\sigma_{s}^{2}+v_{0}^{2}C_{s}^{2}}\right]$$

respectively. Here, $C_{m}$ and $C_{e}$ are sensitivity coefficients of the substrate for mass and elasticity, ρ, μ, λ, and $\sigma_{s}$ are the density, shear modulus, bulk modulus, and the sheet conductivity, respectively of the film, and $C_{s}=\epsilon_{s}+\epsilon_{0}$ ($\epsilon_{s}$ and $\epsilon_{0}$ are the permittivities of the substrate and the medium above it) is the capacitance per unit length of the piezoelectric substrate. It is important to note that pure mass loading effect has also explained by Sauerbrey et al. and employed in BAW sensors [69]. In 1994, Martin et al. advanced the perturbation-based analysis to the case of acoustically thick layers as well [85]. However, other modes of the surface waves rather than Rayleigh wave are more effective for thicker layers [13,14].

**Figure 3:**
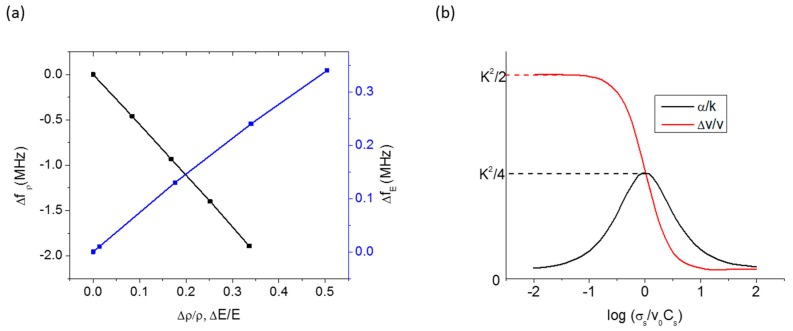
(**a**) Finite element calculation (COMSOL 5.2) of resonance frequency shift of a 128° Y-X LiNbO_3_ SAW transducer as a function of mass density change (Δρ/ρ) and Young’s modulus change (ΔE/E) of a 200 nm thick sensing overlayer (PMMA); (**b**) phase velocity and attenuation as a function of normalized sheet conductivity of a sensing overlayer (Ricco et al. [52]).

The acoustic wave interacts mechanically with mass and elasticity and consequently its velocity changes linearly with them. As given in Equation (3), the velocity shifts negatively and positively with the mass and elasticity loadings, respectively. We calculated the shift in resonance frequency $(\Delta f=f-f_{0}$) due to mass and elasticity changes of an acoustically thin (h=200 nm; Λ~9 μm) and non-conducting Poly-methyl-methacrylate (PMMA) overlayer in a 128° Y-X LiNbO_3_ SAW resonator (*f*_0_ = 436 MHz) using a finite element analysis (COMSOL 5.2) and observed the frequency shifts with the mass and elastic loadings as expected (Figure 3a). The acoustoelectric coupling, on the other hand, causes the mechanical energy to be stored and dissipated so that the resultant sensitivity regime is not necessarily linear. Figure 3b shows a theoretical calculation of the wave velocity and attenuation as a function of the sheet conductivity of an acoustically thin overlayer [52]. From the figure, we see that only a narrow range of the conductivity is active for sensing application.

**Figure 4:**
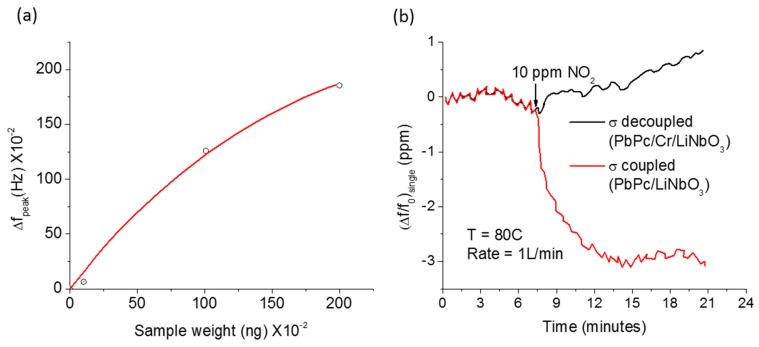
(**a**) Frequency shift of a SAW chemical sensor as a function of loaded mass of *o*-chlrotoluene (Wohltjen et al. [2]); (**b**) separation of mass and acoustoelectric effects of PbPc sensing layer in sensor response to NO_2_ (Ricco et al. [52]).

The mass-loading and the acoustoelectric effect have been extensively explored for SAW chemical sensor development. The first ever reported SAW chemical sensors by Wohltjen and Dessy were based on mass loading [1,2]. They fabricated ST- Quartz and LiNbO_3_ SAW devices with and without sensing overlayers (coatings of greases, squalene, di-n-decyl phthalate, and Carbowax 20 M) and employed those devices as gas chromatography detectors of various polar and non-polar chemicals [2]. They first set up and tested measurement systems for the frequency, amplitude, and phase detection modes and then used the frequency-based technique in an oscillator circuit to detect the presence of various concentrations of different chemicals. A representative result from their work is shown in Figure 4a that shows the frequency shift of a Dow Corning 970 V vacuum grease-coated LiNbO_3_ SAW device upon increasing the mass of *o*-chlrotoluene vapor [2]. Immediately after this study, Muller et al. [86] fabricated SAW devices coated with polyvinylchloride (PVC) and configured as an oscillator for detection of acetone vapor based on the mass-loading. Following these initial reports, several other SAW chemical sensors have been developed in later years using mass loading as the major sensing mechanism [30,57,71,72,73,74,75]. Practical SAW sensors based on conductivity-loading have also been realized and developed for detection and monitoring of variety of chemicals [52,76,77,78,79,87]. In 1985, Ricco et al. [52] experimentally showed for the first time that the conductivity of the sensing layers could also be used as the basis for gas sensing. They fabricated lead phthalocyanine (PbPc) film-coated NO_2_ (in N_2_) SAW sensors on LiNbO_3_ substrates and isolated the effect of the conductivity from that of mass and stiffness to the sensor response using a thin Cr intermediate layer. They observed a large change in the frequency when using the conductivity as the major interaction mechanism whereas almost negligible frequency shift was observed when considering the mass-loading as the major mechanism (Figure 4b). Later, other research groups (e.g., Lec et al. [88], Fisher et al. [89]) have shown theoretically and experimentally that some films can have much larger effect of conductivity change than of the mass and viscoelasticity changes to the wave propagation upon exposure to gases. The development of the chemical sensors using the mass loading and acoustoelectric effect and their sensing applications will be discussed in later sections.

The elastic-loading has been neglected in majority of the developed SAW sensors, that is, it is assumed that the sensing layers’ mechanical properties are minimally altered by a layer-analyte interaction. However, there are some studies that showed significant influence of elastic loading in SAW-based gas sensing, especially when using viscoelastic polymers as the sensing materials [80,81,82,83,84]. In 1992, Grate et al. [81] demonstrated for the first time that the modulus of a sensing layer can be substantially changed upon exposure to the gases so that the elastic-loading can significantly affect the wave propagation. They showed a large effect of swelling-induced modulus of polymer-based sensing layers on the sensor response when exposed to various chemical vapors. In 1995, Falconer et al. developed a grease-coated acetone SAW sensor and explained the sensor response based on the changes in the elastic properties of grease [31]. In the same year, Anisimki et al. evaluated the effect of the change in mass density and the elastic constants of a palladium layer on the surface waves upon exposure to H_2_ gas [80]. In 1996, Cheeke et al. showed a significant contribution of elastic properties of poly-XIOH film when sensing vapors [82]. For a quantitative evaluation of the elastic contribution and its separation from the mass-loading, Hietala et al. proposed a method of using two substrates otherwise identical devices to measure the frequency shift upon exposure to a certain concentration of a gas [90]. They fabricated 97 MHz ST-X quartz and a 100 MHz (001)-cut GaAs SAW sensors with a microporous silicate thin film as the sensing layer and exposed these sensors to dry methanol. They observed a negative frequency change in the quartz sensor indicating a dominant contribution of mass-loading where as a positive frequency change in GaAs sensor indicating a dominant contribution of elastic loading. Using these changes in frequencies with identical sensing layer on different substrates, they were able to calculate the contribution from the elastic loading and the mass loading in each sensor. From the calculation, they found a large change in the modulus of the film upon exposure to the gas, which they explained due to identical size of the pores and the adsorbate molecules. Unlike these studies, Shen et al. observed negligible contribution of the elastic properties of polyaniline (PANI) compared to the mass-loading when sensing NH_3_ using a SH-SAW sensor [83]. More recently, Raj et al. fabricated a ZnO-coated SAW NH_3_ sensor and studied the relative contribution of electric, mass, and elastic loadings to the frequency shift [84]. They observed a dominant contribution from the mass-loading and the elastic-loading whereas the acoustoelectric effect had a very small contribution to the sensor response. Development of practical SAW chemical sensors based on the viscoelastic properties requires further investigation.

## 3. Sensor Characteristics

The characteristics such as sensitivity, selectivity, response kinetics, detection limit, repeatability, and stability of a SAW chemical sensor depends upon the materials properties, device design, fabrication techniques, operation frequency, and environmental conditions [91,92]. Piezoelectric SAW transducers in principle offer ultra-high sensitivity, fast response, suitable size and structure, and compatibility with other technologies. However, the sensitivity, selectivity, response times, and other factors determining the suitability of SAW sensors for an application depend critically on the properties of the sensing layers [23,92].

**Figure 5:**
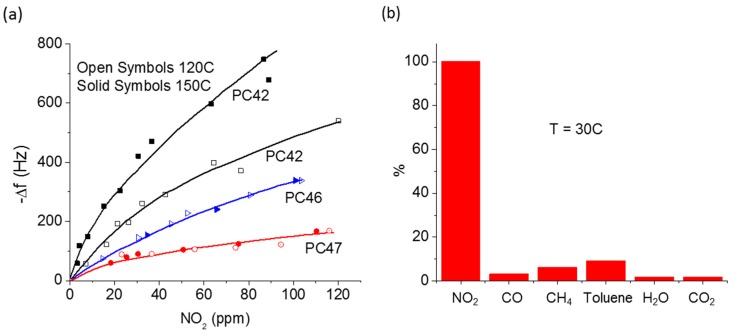
Sensitivity and Selectivity of SAW gas sensors (Venema et al. [72]).

SAW sensors can detect chemicals at very low concentrations (~ppb level). The sensitivity (variously expressed in Hz/ppm, Hz/vol%, or deg/ppm) depends upon the piezoelectric transducer properties, the center frequency, the sensing layer properties (e.g., material properties, thickness, and surface roughness), and temperature of operation [19,23,30,75,93]. Several studies have shown that their mass sensitivity (Δf/Δm) increases as the square of the operating frequency [30,75,93]. For example, Dickert et al. experimentally showed a parabolic increase in sensor sensitivity and a linear increase in noise level with center frequency when going from 80 MHz to 1 GHz [93]. Thickness of the sensing layers is also found to be a major factor to affect the sensitivity of SAW sensors [72]. In ideal cases, the sensitivity increases with thickness but changes in roughness, crystallinity, and hardness of a layer with thickness have potential impact in the sensitivity [72]. For instance, an increased roughness attenuates the acoustic waves thereby decreasing the sensitivity of the sensors. The sensitivity is also greatly affected by the substrate temperature. Venema et al. fabricated LiNbO_3_ SAW sensors of different operating frequencies, coated metal-free PC films with three different thicknesses, and operated at two temperatures for detection of NO_2_ gas (Figure 5a) [72]. For a given thickness of the sensing layer, they observed higher sensitivity of the sensor operating at higher frequency. However, the sensor operated at lower frequency and containing thinner overlayer was more sensitive than the one operated at higher frequency and containing thicker layer. Similarly, they observed higher detection sensitivity to NO_2_ at higher temperature.

The selectivity of SAW chemical sensors is solely dependent upon the material properties of the sensing layer [16,76]. When a SAW sensor is exposed to a mixture of various gases, it sorbs or reacts with them differently so that the strength of the sensor output is different for each component. Venema et al. [72] employed the PC-coated sensors (described above) for selective detection of NO_2_ in a mixture of various gases, the results of which are shown in Figure 5b. This figure clearly shows that the SAW sensor coated with a proper material can be used for selective detection of analytes. There is a large volume of studies concerned with finding optimal materials to use as the sensing layer in SAW sensors for selective detection of various gases and chemicals [16,76,92].

In addition to the selectivity, the reversibility and repeatability depend upon the materials properties of the sensing layer and its interaction type with the analytes. If the interaction is by physisorption, there is a high chance of a reversible and repeatable response provided sufficient time is allowed for equilibration but this type of interaction leads to a less selective detection. On the other hand, if the interaction is by chemisorption, the absorption is relatively more selective but has relatively poor reversibility due to the formation and breaking of chemical bonds. The stability and hence the reproducibility of the sensors depend upon the stability of the used materials and the compatibility between the sensing material and the piezoelectric substrate. If the mechanical adhesion between the substrate and the sensing layer is strong and the film does not deform, the sensor can last for longer time [7,92].

The rate of layer-analyte interaction and hence the response time of a SAW sensor is affected by several factors. In the case of mass-based sensing layers, the response and recovery times of a SAW sensor mainly depend upon the rate of diffusion of the adsorbed mass into the film and to the piezoelectric substrate and back to the film surface. The sensors with thinner sensing layers usually have faster response kinetics. With decreasing the thickness, the gas diffuses in and out of the film rapidly so that time required to reach to the equilibrium decreases. Thin sensing layers thus result in rapid response to gases [72,89]. In a frequency-dependent study, Dickert et al. have shown that the sensors with higher operational frequency can have shorter response time in addition to their improved sensitivity [93]. They attributed the reduction of the response time to the possibility of using thinner layers in the high frequency transducers. It has also been shown that the response time of a SAW chemical sensor varies with temperature [72,94]. With increasing the temperature, the diffusion/dissociation rate of the analytes may increase thereby decreasing the response time.

## 4. Advances in SAW-Based Chemical Sensing

In recent years, there have been impressive advances in various aspects of SAW chemical sensors (e.g., [4,13,78]) even though early studies were focused in understanding the sensing mechanism and exploring the detection techniques [2,3,19,22,25,30,33,52]. Major directions of the advancement include (i) the optimization of sensing layer properties and coupling of the layers with analytes and piezoelectric substrates [91]; (ii) the control of the undesired effects and drifts that can arise from the devices itself, the environment, and the measurement system [91]; (iii) application for detecting and monitoring a range of gases, VOCs, and CWAs at different operating conditions [66,95]; and (iv) integration with electronics and improvement in signal analysis. Below, we discuss some of these advances.

### 4.1. Sensing Layer Considerations

The properties of the sensing layers and their coupling with the target analytes and piezoelectric substrates are important factors to consider for developing high performance sensors [96]. A good sensing material must be able to interact with the target gas selectively, strongly, and quickly while maintaining its stability over time. Selective detection of gases can become challenging as a sensing material can interact with multiple gases (or many sensing materials can interact with the same gas as well) [97]. However, tuning the sensor response is possible by selecting proper materials or their derivatives for specific analyte types and targeted applications [16]. In addition, an ideal overlayer has to have reversible and repeatable interaction with the analytes, strong mechanical adhesion with piezoelectric substrates, be tolerant to the measuring environment, and leave IDTs open electrically [17]. In the past, the focused efforts to improve the couplings include (i) selection of the materials with high sorption capacity and high adhesion; (ii) control of the physical parameters (thickness, uniformity, roughness etc.) of the layer; and (iii) use of optimal coating techniques [17,89,97].

A range of materials have been tested as the sensing layers for SAW-based gas sensing [16,96,98]. Most commonly used materials are conducting/non-conducting polymers and semi-conducting metal oxides [92,94]. Other widely used materials include carbon nanostructures (e.g., carbon nanotubes [99,100,101] and graphene oxide [77]), metals [102,103], ceramics [78], and composite materials [101,104,105]. More recently, interest is in relatively new materials such as metal-organic-frameworks (MOFs) [106] and porous materials for their higher sorption capacity [107]. These materials are applied using different techniques such as sputtering, evaporation, spin coating, drop casting, spray coating, and Langmuir-Blodgett methods depending upon the properties of the materials [16,108]. To fulfil the requirement of particular sensing application, materials with suitable material properties and proper interaction type with analytes have to be selected as a sensing layer [95,97,109].

Different types of conducting/non-conducting polymers and their derivatives have been used for developing room temperature chemical sensors for detection of various VOCs such as alcohols, aromatic compounds or halogenated compounds, and some inorganic gases. Some widely used variants include pure polymers, functional organic polymers, molecularly imprinted polymers, self-assembled polymers, and polymer composites [16,110]. The sensors based on polymer films have usually short response and recovery times as they generally absorb the gases via physisorption. Redox reactions can be involved in case of conductive polymers, especially when they are doped to increase the conductivity. Another advantage of polymers comes from the ease of fabrication. They are solution processable and can be coated by simple techniques such as spin coating and drop casting. The selectivity of the sensors based on these materials is relatively poor even though it can be enhanced to some extent by some modification in their structure or by doping.

Metal-oxides, on the other hand, have been widely used to detect inorganic gases and some VOCs at high temperatures [16,97]. Some popularly used metal oxides in SAW sensors include the films and nanostructures of WO_3_, In_2_O_3_, ZnO, SnO_2_, TiO_2_, TeO_2_, and Co_3_O_4_ [78,94]. Their high temperature suitability comes from the fact that they (i) possess high thermal stability; and (ii) mostly interact with gases at elevated/high temperatures. These materials interact with different oxidizing and reducing gases via chemisorption or redox reaction depending upon the temperature [96]. This helps developing selective sensors using these materials. The sensitivity of these materials is weak at room temperature but can be tuned at higher temperatures depending upon the materials and target analytes. However, they may exhibit very long recovery time due to the involvement of redox reactions that limit their use for real time gas monitoring and sensor array development.

Other materials such as carbon-nanotube [99], graphene [111], and a variety of composite materials [99,100,101] have attracted recent attention for their potential to overcome the limitations of polymers and metal-oxides [16,98]. For instance, carbon nanotubes have shown very high sensitivity to various VOCs and inorganic gases at room temperature. They have large surface-to-volume ratio, fast response time for many gases, and high adsorption capacity that make them promising materials for development of sensitive room temperature sensors [98]. They can also be decorated with other materials to improve the sensitivity and selectivity. Composites of semiconductors (SCs) and metals have also shown improved sensitivity and response time. Ippolito et al. developed H_2_ SH-SAW sensors using pure and Au catalyzed WO_3_ (by Au and Pt) as sensing layers on ZnO/LiTaO_3_ layered piezoelectric substrate [112]. They were able to detect 0.06% H_2_ in air using the catalyzed sensors whereas 0.5% H_2_ was the lowest limit for pure WO_3_ sensor at different temperatures.

**Figure 6:**
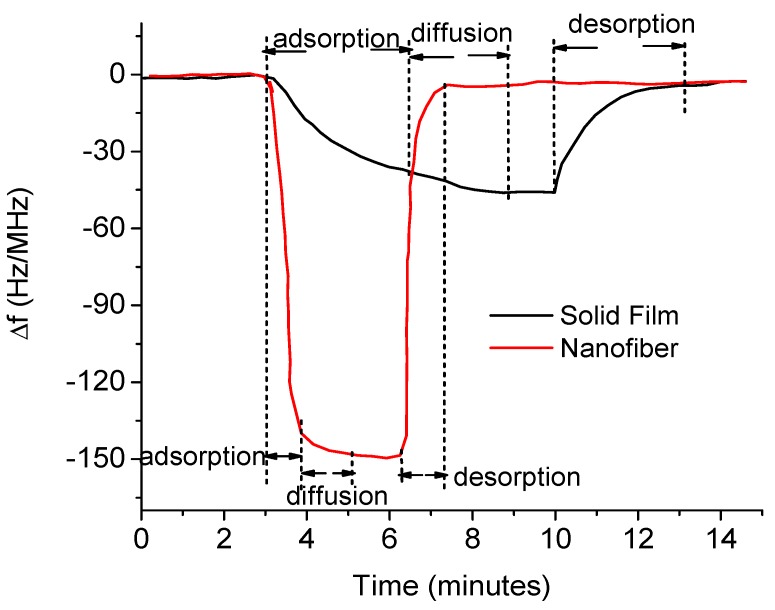
Response of a SAW sensor coated with nanofibrous and solid films of polyethylene oxide to hydrogen peroxide gas (Liu et al., [116]).

Recent reports have shown that using nanostructures (e.g., nanorods, nanofibers) rather than solid films can improve the sensing response of polymers and metal oxides as well [113,114,115]. As an example, Sadek et al. [104] fabricated a layered ZnO/60° Y-X LiNbO_3_ sensor, functionalized the surface with polyaniline/In_2_O_3_ nanofiber composite, and employed for detection of H_2_, NO_2_, and CO at room temperature. They observed faster response and recovery times compared to those of a solid film-based otherwise identical sensor with a good repeatability. Similarly, Giffney et al. [113] fabricated identical LiNbO_3_ SAW sensors using nanorods and spin-coated films of ZnO as the sensing layers and employed for ethanol vapor detection at 270 °C. They showed a large frequency shift (24 kHz) in the nanorod-based sensor against a shift of 9 kHz in the film-coated sensor when exposed to the same concentration (2300 ppm) of ethanol vapor. Liu et al. showed that the nanofiber-based SAW sensor can have much higher sensitivity, fast response, and shorter recovery time compared to those of a solid film-based identical sensor [116]. Figure 6 is a result obtained by them for detection of 30% hydrogen peroxide (H_2_O_2_) in air using a ST-X Quartz SAW sensor coated with nanofibrous and solid films of polyethylene oxide. Given additional advantage that these nanostructures can be fabricated and coated using simple experimental techniques such as electrospinning, solution growth, and sol-gel; they are promising materials for sensitive sensors.

Besides exploring various materials for sensing layer, there have been studies to optimize the physical parameters of the layer such as the thickness, uniformity, surface roughness, and its mechanical adhesion with substrate to improve the sensor response [94,97,107]. In 1986, Venema et al. [72] showed the highest sensitivity of a SAW sensor to NO_2_ with the thickest PC-coating. However, later it became clear that there exists a critical thickness of the layer beyond which the sensitivity starts decreasing. For instance, Luo et al. [94] studied the effect of the film thickness (150 nm–382 nm) of SnO_2_ film on the sensitivity and response time in detecting H_2_S. They observed the highest sensitivity and the shortest response time with the film thickness of 275 nm suggesting the importance of choosing an appropriate thickness for optimal sensor response. In 2010, Fisher et al. showed large changes in propagation loss of Pd-coated Y-Z LiNbO_3_ SAW delay line depending upon the film thickness and suggested a need of further study in understanding the effect of film thickness on SAW gas sensing [89]. On this basis, Tasaltin et al. showed that the dominant interaction mechanism between the ZnO nanostructured sensing layer and SAW waves can change from acoustoelectric to mass/elastic effect beyond a certain thickness [78].

Some researchers have reported a possibility of improving the sensitivity of SAW chemical sensors by designing metal/SC layered sensing coatings instead of using a metal-only or SC-only layer [77,112,117,118]. For example, Jakubik et al. developed LiNbO_3_ SAW sensors for H_2_ detection by coating copper PC (CuPc), Pd, WO_3_, and their combinations to form metal/SC layered structures [13,118,119]. They observed a much higher sensitivity to H_2_ gas when using a metal/SC layered structure compared to the single metal or SC sensing film. The improved sensitivity of the bilayer-based sensors over metal or SC-only layer was attributed to an extended active conductivity regime of the layered structure. The metal-only layer shorts the electric field associated with the SAWs causing only mass-loading to be effective as the interaction mechanism whereas the SC-only layer could result to a weak acoustoelectric coupling due to relatively small active regime of the conductivity (Figure 3b). However, when metal/SC layered structure is formed, the active conductivity regime and hence the acoustoelectric coupling between the layers and surface waves can be extended to a higher sensitivity range. This leads to the detection of target gases with higher sensitivity.

Selection of a proper technique to apply sensing layers on SAW transducers is another important factor to consider for improving the sensor performance. For instance, a polymer film obtained by dip coating or by layer by layer deposition can have well controlled thickness, smooth surface, and better adhesion with the substrate than the one obtained by spin coating or drop casting. McGill et al. [108] have made a comparative study of various chemoselective polymers and different deposition techniques for optimal response of SAW chemical sensors. Similarly, Pestov et al. showed an improvement in stability of the sensing layers using photo-polymerization on SAW resonators [120].

### 4.2. Controlling the Interfering Factors

In spite of their high sensitivity to chemicals, SAW sensors may suffer interferences from various internal and external factors so that the output may deviate from the reality [121]. The internal factors that may cause spurious response include the viscoelastic properties of the sensing layers, wave reflections from the edges of the devices, impedance mismatch of the IDTs, and the temperature rise of the substrate materials during RF signal excitation. The external factors that may cause a deviation of the sensor response include the environmental factors such as humidity, temperature, pressure, and the presence of unwanted chemicals. Finally, measuring instruments and signal analysis could also introduce errors in the sensor output. Different techniques have been suggested and employed to minimize various possible drifts in these sensors [72,121,122].

The unwanted effect from the sensing layers such as the viscoelasticity, incompatibility, roughness, etc. can be controlled by selecting robust sensing materials or using better deposition techniques [16,108,120]. Similarly, the reflections from the edges of the devices and IDT impedance mismatch can also be addressed to some extent by using wave absorbers at the devices edges or optimizing the IDT and reflector designs (e.g., [123]). The temperature rise due to temperature dependence of the piezoelectric substrates, the sensing materials, or IDT materials can be addressed by using less temperature sensitive materials. As noted in Table 1, the piezoelectric crystals with selective orientations have low TCF that provides more stable sensor signal with reduced cross-sensitivity to temperature.

**Figure 7:**
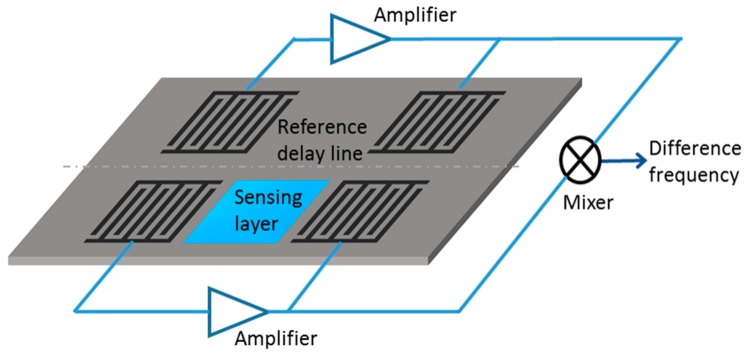
Schematic of a dual delay line SAW sensor for compensation of thermal drift.

The sources of the thermal drift are not only the materials but also the environment. The environmental thermal drifts can affect the materials properties of the substrates and the sensing layers such as the dielectric constants, conductivity, and elasticity that have direct influence on the wave propagation. To compensate the thermal drifts from the environment or from the piezoelectric substrates, researchers often use a reference device in the same substrate (i.e., dual delay line devices) [8,52,57,72,124]. Figure 7 shows such a schematic of a dual delay line oscillator (differential scheme) to compensate the thermal drifts in which one delay line is coated with the sensing material of interest for gas exposure and the second delay line is left uncoated. The signal in the unloaded delay line can be subtracted from the loaded delay line signal to remove the thermal and other environmental drifts. This scheme has been useful in compensating the thermal drifts from the substrate itself or from the environment. However, it is unfit to address the temperature rise during layer-analyte bonding or any other factors associated with the sensing layer [57].

Another important source of the environmental drifts is humidity. Several studies have shown that humidity may cause an increase or decrease of the detection sensitivity depending upon the material properties [78,125]. In 1993, Rebikre et al. pointed out that a CuPc-coated NO_2_ SAW sensor can have higher sensitivity in more humid environment [125]. In a recent study by Tasaltin et al., the humidity was found to have both positive and negative effect to the sensitivity of a ZnO-coated SAW sensor [78]. They observed an increase in the sensitivity for acetone, chloroform, propanol and trichloroethylene vapors with humidity while a decrease for methanol and ethanol vapors. The effect of humidity on sensor response can be mitigated operating the sensors at higher temperatures [126]. Besides operating at higher temperatures, using a hydrophobic material as the sensing layer can also be an effective approach to avoid the humidity effect.

In addition, there can also be other sources of drifts including the measurement system itself and the remanence of a previous measurement. The drifts or instability from the measurement system can be addressed using high resolution instruments and careful integration of devices with the electronics. Similarly, one can eliminate the drift from the remanence by designing a measurement system for complete removal of the residuals. For instance, Muller et al. demonstrated a cyclic measurement technique to remove such drifts in detection of NO_2_ using SAW sensor [127].

More recently, there has been effort to addressing various drifts collectively. For example, Wen et al. [128] fabricated a dual-track SAW sensor with WO_3_ coating that showed a good response characteristics to small concentrations of NO_2_ gas. They were able to detect down to 0.5 ppm of NO_2_ gas with good reproducibility and stability. They claimed that this configuration could eliminate the external perturbations, suppress the bulk waves, and improve the side lobe rejection thereby improving the sensor response. Nevertheless, there are needs to minimize the device cross-interferences to several parameters including temperature, strain, unwanted chemicals, and humidity for their efficient use as chemical sensors.

### 4.3. Applications

SAW device platform is very flexible for virtually any analyte through optimized integration with sensing layers. Numerous gases, chemical vapors, and CWAs have been detected and monitored using this platform. Early SAW sensors based on mass-loading (e.g., Bryant et al. [28,91], D’Amico et al. [103], Venema et al. [72]) were developed for detection of gases such as H_2_, SO_2_, and NO_2_. Bryant et al. [28] detected SO_2_ gas using a set of SAW sensors in 1981 and compared their detection sensitivity with that of BAW sensors. They found that the SAW chemical sensors were at least an order of magnitude more sensitive (able to detect less than 70 ppb of SO_2_) than the corresponding sensors based on BAWs. D’Amico et al. [103] developed the first H_2_ SAW sensor in 1982 by coating a palladium (Pd) sensing layer on a 76 MHz Y-Z LiNbO_3_ to operate at room temperature. In 1986, Venema et al. showed selective detection of NO_2_ from a mixture of gases using a polymer-coated SAW sensor [72]. Similarly, the sensors based on conductivity loading (e.g., Ricco et al. [52], Lee et al. [129], and Fisher et al. [89]) were also used for detection of various gases including H_2_, SO_2_, and NO_2_. Lee et al. developed a 54 MHz LiTaO_3_ dual delay line SAW sensor in 1998 by coating a cadmium sulfide (CdS) film for monitoring SO_2_ gas [129]. This sensor gave a frequency shift proportional to the SO_2_ concentration which was explained based on the sensing layer’s mass and electric field changes upon exposure to the gas. In 2010, Fisher et al. developed a H_2_ SAW sensor using a Pd-coating and studied the influence of the acoustoelectric, mass, and stiffness effects on to the SAW velocity and attenuation [89]. They found the sensor velocity and attenuation were substantially dependent on the conductivity change of the film rather than the mass and the elastic changes upon exposure to H_2_ gas.

Over time, many SAW sensors based on Rayleigh waves have been developed for detection of a range chemicals (in gaseous phase) including H_2_ [102,103], H_2_S [31,94], SO_2_ [129], N_2_ [52], NO [105], NO_2_ [111,127,130], NH_3_ [131,132], CO [104], CO_2_ [32,130], CH_4_ [133], O_2_ [29,134,135,136], and O_3_ [137]. Similarly, humidity [9], and many organic vapors such as methanol [138], ethanol [139], acetone [31], styrene [140], dimethyl methylphosphonate (DMMP) [95] have also been detected using these sensors. With suitable polymer coating, the sensors have also been employed for detection of various explosive chemicals [141,142]. Many research teams have worked in developing the SAW sensor arrays for simultaneous detection of chemicals [95,130,138,139,143,144,145]. Advances have been made to operate the sensors at high temperatures and harsh environments as well [29,146,147,148,149]. Table 2 lists some SAW sensors from the literature for detection of various gases or chemical vapors at different conditions. Below, we discuss some advances of these sensors for harsh environment and sensor array applications.

#### 4.3.1. SAW Sensors for Harsh Environment

**Figure 8:**
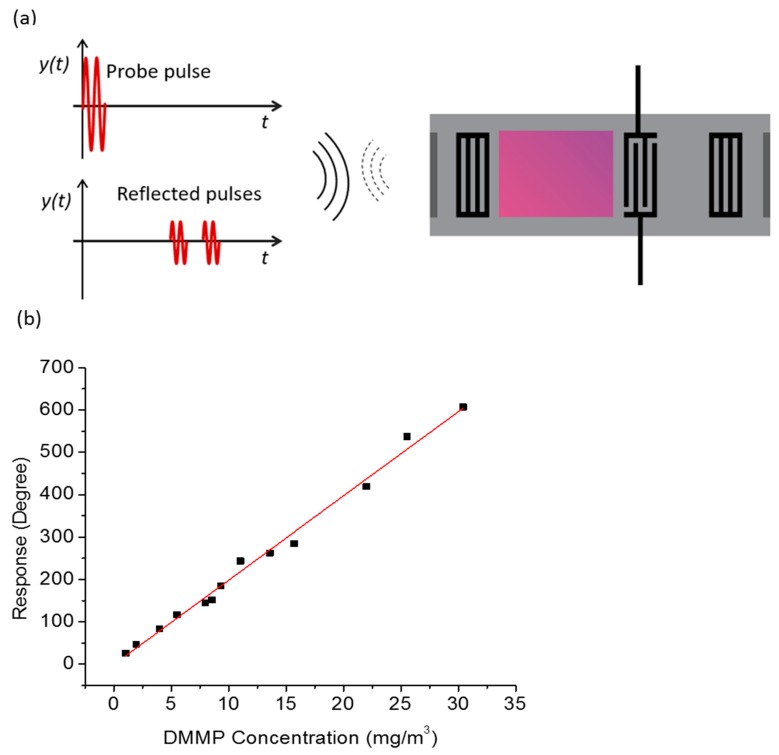
(**a**) Schematic of a wireless SAW sensor (Greve et al. [29]) and (**b**) response of a fluoroalcoholpolysiloxane (SXFA)-coated wireless SAW sensor to various concentrations of DMMP (Xu et al. [57]).

One important advantage of SAW sensors is their ability to work in wireless mode. They can work as passive transponders in which the acoustic waves can be excited and received remotely using RF electromagnetic signal (Figure 8a). This ability makes it possible to operate the SAW sensor in extreme conditions such as high temperature, high pressure, and toxic environment. This can be beneficial for detecting chemicals produced in automobiles, combustion engines, mine and oil industries. There has been some progress in detecting different gases such as CO_2_, NH_3_, NO_2_ using wireless SAW sensors such as those by Thiele et al. [148], Lim et al. [130], and Wen et al. [67]. In 2007, Wen et al. [67] developed a wireless SAW reflective delay line with 440 MHz central frequency, coated with Teflon AF as a sensor layer, and demonstrated detection of CO_2_ gas remotely using a vector network analyzer. They found a sensitivity of 1.98°/ppm with good linearity and repeatability in 0–450 ppm concentration range. They were also able to investigate the effect of temperature and humidity in sensing CO_2_ in wireless mode. In 2011, Lim et al. fabricated a passive sensor of similar configuration and employed for detection of CO_2_ and NO_2_ remotely while measuring the temperature simultaneously [130]. Similarly, Xu et al. [57] fabricated and employed a reflective delay line wireless SAW sensor for detection and monitoring of organophosphorous compound vapors at 25 °C (Figure 8b). They were also able to study the effect on the sensitivity of the wireless sensor when increasing the temperature to 80 °C.

Regardless the use of wireless mode, there are several other challenges of developing SAW sensors for harsh environment operation. These include the degradation of materials, unwanted chemical reactions in the operating environment, and need of antennae for efficient communication between the devices and measuring instruments. Some research groups have developed SAW sensors using relatively more stable materials at higher temperatures. For instance, Greve et al. [29] developed O_2_ SAW sensor to operate at harsh environment using langasite substrate, Pt electrodes, and SnO_2_ sensing layer. They were able to detect 10% O_2_ in N_2_ at 650C using wired mode operation.

#### 4.3.2. SAW Sensor Array

**Figure 9:**
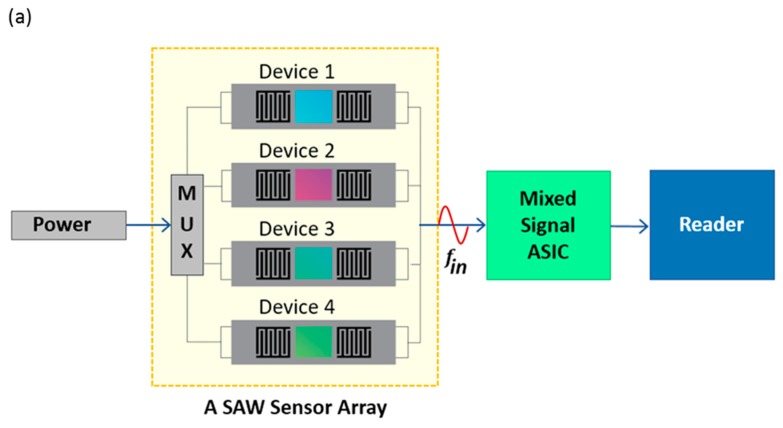

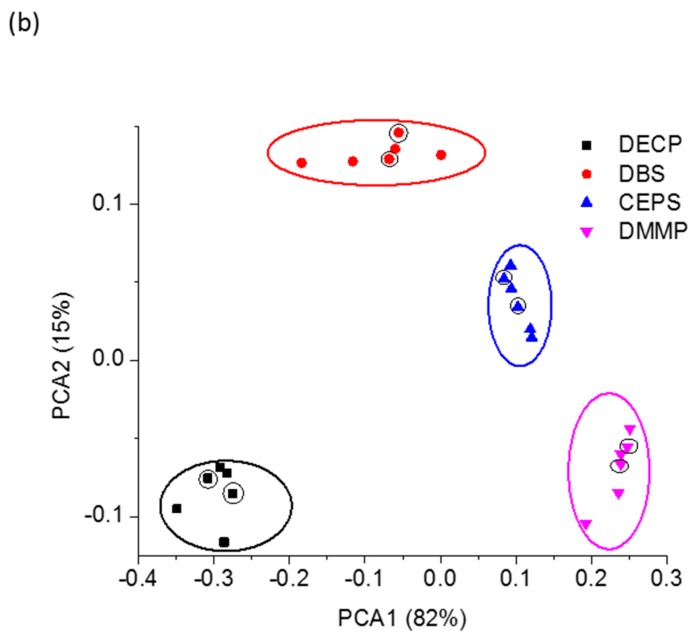
(**a**) Schematic of a SAW sensor array (Tang et al. [139]); (**b**) Electronic nose application of a SAW sensor array in combination with principal component analysis to discriminate various vapors (Raj et al. [169]).

SAW chemical sensors can be developed for selective detection of a gas in a mixture as well as simultaneous detection of various gases [143]. When a SAW sensor is coated with a material sensitive to a particular gas and exposed to a gas mixture, it is possible to detect the intended gas selectively. Many research groups have developed selective gas sensors coating specific sensing material on SAW transducers. For example, Penza et al. [131] fabricated a polypyrrole-coated SAW resonator to detect NH_3_ in a mixture of NH_3_, CO, CH_4_, and H_2_. They found a very good sensitivity to NH_3_ gas whereas the sensor was poorly sensitive to other gases. On the other hand, an array of SAW sensors on a substrate can be used to detect the analytes simultaneously and discriminate them from each other (E-nose application) when each device in the array are coated with different sensing materials [95,130,138,139,143,144,163,164,165]. Figure 9a shows a schematic of a SAW sensor array for simultaneous detection of various gases [139].

In 1995, Frye et al. [166] developed a SAW sensor for in situ detection and monitoring of a range of VOCs using polymer sensing layers. In 2000, Raap et al. [167] developed a miniaturized SAW-sensor array driven by multiplexed oscillators for organic gas detection. Later in 2003, Ho et al. [168] developed a SAW array device with polymer coatings for in situ detection of a wide range of VOCs. These sensors did not require carrier gas and were operational in any media (air, soil, or water) unlike the VOC sensor developed by Frye et al. In 2007, Joo et al. [109] fabricated a polymer coated SAW sensor array and employed them to classify CWAs. Specifically, they fabricated a sensor array using five SAW sensors coated with polyisobutylene (PIB), polyepichlorohydrin (PECH), polydimethylsiloxane (PDMS), polybutadiene (PBD) and polyisoprene (PIP) and employed the array to detect DMMP, acetonitrile (CH_3_CN), dichloromethane (CH_2_Cl_2_) and dichloropentane (DCP) CWAs. They could detect down to 5 ppm of the CWAs with good selectivity. In 2011, Matatagui et al. [95] developed an E-nose based on polymer-coated SAW sensors for detection of various CWAs. They were able to detect very low concentration, namely 0.05 ppm of Dimethyl DMMP and 0.5 ppm of dipropyleneglycol monomethyl ether (DPGME) using an optimized sensor array.

The capacity of an E-nose sensor to discriminate various chemicals can be better expressed using principal component analysis (PCA), a statistical data analysis technique [169]. Figure 9b shows a PCA analysis of the response of a metal-oxide SAW sensor array to various CWAs as obtained by Raj et al. [169]. They fabricated a SAW sensor array using ZnO, TeO_2_, SnO_2_ and TiO_2_ and employed for selective detection of various CWAs. They obtained a good sensitivity of the E-nose to sub-ppm level concentrations of DMMP, dibutyl sulfide (DBS), chloroethyl phenyl sulfide (CEPS) and diethyl chlorophosphate (DECP). A PCA analysis of the differential frequency shift of the sensor outputs showed high capacity of the E-nose in discriminating the CWAs (large ellipses in Figure 9b). They could define different regime in the PCA graph for the stimulants using the frequency shift for four concentrations. The frequency shift obtained for other concentration of specific stimulant was shown to fall in the defined regime (small circled points in the ellipses in Figure 9b) for that stimulant indicating an efficiency of SAW E-nose in discriminating various chemicals.

## 5. Summary and Outlook

We have discussed various aspects of Rayleigh SAW chemical sensors that include the working principle, factors interacting with SAWs, and progress in detecting various chemicals in gaseous state. Many SAW chemical sensors have been developed to date including mass-loading and acoustoelectric effects of overlayers for detection of inorganic gases, organic vapors, and chemical warfare agents amongst others. While mass-loading effect allows for a simpler transduction mechanism, the ultimate sensitivity is limited by the amount of mass uptake that can be accomplished for a specific sensing layer design. In contrast, the acoustoelectric effects allow for a significantly larger overall sensing response and hence sensitivity in many cases but requires the conductivity of the sensing layer to be engineered within an optimal range. Only a limited number of SAW based chemical sensors have been developed based upon the viscoelastic properties of sensing layers due to the need for engineered sensing layers with relatively large changes in viscosity resulting from analyte interactions. To improve the sensor characteristics, numerous research groups have tested a variety of materials to be applied as piezoelectric substrates, IDT electrodes, and sensing overlayers. In some cases, these sensors have been demonstrated to show wireless operation and in other cases, studies were focused on simultaneous detection of chemicals using an array of the sensors. A relatively limited number of works have also begun to explore this class of sensor devices for chemical sensing in extreme environmental conditions for which the stability of the underlying sensor platform as well as the electrode materials and functional sensor layers become an important consideration. Also, there is a need of designing and integrating high capacity antennae for these devices to operate remotely with minimal loss of energy when considering harsh environment applications.

Due to the inherent advantages of the SAW-based sensor platform, it is anticipated that functionalization of SAW devices for chemical sensing will continue to be a highly-investigated area of research moving into the future. Potential future research directions on SAW-based gas sensing include: (i) continued exploration of novel sensing materials to advance the performance and applications; (ii) advancement of passive and wireless sensors for high temperature and harsh environment gas sensing; and (iii) development and demonstration of multi-element SAW sensor arrays for multi-component gas analysis specification and quantification. From a materials perspective, primary areas of research need and interests include (i) development of electrical conductor materials for reflectors and interdigitated electrodes with stability at high temperatures in relevant gas atmospheres; (ii) functional sensor materials with high temperature stability and controlled, reversible interactions with analytes of interest; (iii) sensing materials with engineered high total sorption capacities such as through nanostructuring; (iv) sensor layers with tunable absolute electronic conductivities and engineered conductivity responses associated with targeted analyte interactions; and (v) exploration of novel materials with relatively large and useful viscoelastic responses to analytes of interest upon gas absorption. Development of flexible SAW chemical sensors using novel piezoelectric substrates could be another potential research direction.

## Figures and Tables

**Table 1:** Some common piezoelectric materials for SAW chemical sensors. An (*) is for a SAW other than Rayleigh mode and a (^†^) is for a measured velocity.

Substrate Material	Reported SAW Velocity (m/s)	*K*^2^ (%)	TCF (ppm/C)	ε	T_max_ (C)
ST-X Quartz	3159.3 [30]	0.11	0	3.7	573
Y-Z LiNbO_3_	3487.7 [30]	4.80	94		1150
128Y-X LiNbO_3_	3992 [43]	5.6	75	83	
64Y-X LiNbO_3_	4742.5 * [54]	11.3	80		
Y-Z LiTaO_3_	3230 * [42]	0.74	35		665
X-112Y LiTaO_3_	3301 * [45]	0.64	18	52	
(0, 138.5, 26.8) La_3_Ga_5_SiO_14_	2734 ^†^ [44]	0.34	~0	18	1470
(0001) AlN	5607 [55]	0.30	19	8.5	2200
(001)-<110> GaAs	2864 [52]	0.07	35	12.9	
ZnO	2645 [47]	1.8	15	10	1170

**Table 2:** Selected published work in SAW delay lines (DL) and resonators (Res) for sensing various chemical vapors and gases.

Analyte	Transducer Detail	Sensing Layer	Sensitivity (η) and Lower Limit (LL)	Operating Condition/Comments
H_2_	75 MHz Y-Z LiNbO_3_ 2-port DL [103]	Palladium (Pd)	LL = 50 ppm, phase	H_2_ in N_2_ at room temperature (@RT)
	12MHz 128° Y-X LiNbO_3_ 2-port DL [150]	Sputtered InO_x_	η = 11.83 kHz/400 ppm (H_2_ in air), LL = 100 ppm H_2_ mixed with N_2_	H_2_ in N_2_ or air, @RT, 55% RH, measured *f*, ϕ, and insertion loss IL (ϕ and IL not stable below 2000 ppm)
	107.2 MHz ZnO/64° Y-X LiNbO_3_ 2-port Res [151]	Polyaniline/WO_3_ composite nanofiber	η = 7 kHz/1%H_2_, LL = 0.06%	H_2_ in synthetic air @RT
H_2_S	60 MHz Y-Z LiNbO_3_ 2-port DL [152]	Sputtered WO_3_	η = 0.35 kHz/ppm, LL < 1 ppm	H_2_S in air @130 °C
	147 MHz 36° Y-X LiTaO_3_ 2-port DL [153]	SnO_2_/CuO by sputtering	η ~ 16.9 kHz/ppm, LL ~ 0.53 ppm	H_2_S in air, @160 °C (70–205 °C), SH-SAW sensor
	118.5 MHz 64° Y-X LiNbO_3_ 2-port DL [100]	Cu NP-decorated SWCNT/drop-cast	LL = 5 ppm	H_2_S, H_2_, ethanol, acetone in air, @RT and 25–200 °C, stable f = 1 Hz
NO_2_	ZnO/SiO_2_/Si and Quartz DL [154]	CuPc by physical vapor deposition (PVD)	η ~ 920.0 Hz/ppm	NO_2_, NH_3_, and H_2_O @ 150 °C; variable sensitivity
	101.764 MHz; 128° Y-X LiNbO_3_ [128]	Sprayed WO_3_	η ~ 7 kHz/ppm, LL = 0.5 ppm	Dual track SAW device, @25 °C, 80 °C
	262 MHz ST-X Quartz 2-port Res [111]	Graphene by ink-jet printing	η ~ 25 Hz/ppm, LL < 0.5 ppm	NO_2_ in air, @RT
CO_2_	440 MHz 41° Y-X LiNbO_3_ reflective DL [32]	Teflon-AF	η ~ 2°/ppm	@RT, humidity and temperature effects
	250 MHz ST-Quartz 2-port Res [155]	Spin-coated polymers	η ~ 4.17 Hz/ppm	CO_2_ in N_2_@RT, CO_2_ and H_2_O vapor studied, third harmonic analysis of central frequency was performed
	286 MHz 128° Y-X LiNbO_3_ DL [156]	Self-assembled functionalized SWCNT	η ~ 6 mV/% (attenuation), LL ~ 3.5%	CO_2_ in N_2_ @RT, studied the humidity effect
CH_4_	363 MHz Y-Z LiNbO_3_ DL [157]	Sputtered SnO_2_ (for CH_4_)	LL < 500 ppm	Contactless sensor, CH_4_, NO_2_, toluene in air, @300–450 °C.
	299.4 MHz ST-X quartz 2-port Res [133]	Spin-coated or drop-casted Cryptophane-A	η ~ 204 Hz/%, LL ~ 0.05%	CH_4_ in N_2_, humidity effect, @RT
SO_2_	131 MHz AT-cut Quartz DL [158]	Sprayed triethanolamine (TEA)—boricacid composite	η ~ 200 Hz/ppm, LL < 8 ppm	SO_2_ in N_2_@12 °C, used TEA, TEA-boric acid composites, and boric acid as sensing layer
	54 MHz LiTaO_3_ DL [129]	Spray pyrolysis of CdS, mass and electric loading	LL < 200 ppb	SO_2_ in air @RT
NH_3_	100 MHz 128° Y-X LiNbO_3_ DL [159]	Brushed-coated l-glutamic acid hydrochloride	η ~ 0.48 ppm/ppm LL = 0.56 ppm	NH_3_ in air @RT, (frequency-based), humidity effect
	42 MHz 128° Y-X LiNbO_3_ DL [131,160]	LB-coated polypyrrole	η ~ 0.13 ppm/ppm	NH_3_ in mixture of CO, CH_4_, H_2_, O_2_ @RT, (phase-based)
	ST-cut Quartz Res [132]	ZnO/SiO_2_, sol-gel/spin-coated	η = 66.7 Hz/ppm LL= 5 ppm	NH3 in air, @RT
SF_6_	42 MHz 128° Y-X LiNbO_3_ DL [161]	Drop cast of acid-treated MWCNT	η = 7.4 kHz/ppm, LL = 9.5 ppm	SF_6_, SO_2_, and HF in air @RT, (dual track SAW)
O_3_	433 MHz Y-Z LiNbO_3_ reflective DL [137]	Spin-coated Polybutadiene	LL = 63 ppb	O_3_ in dry air @RT, recorded temperature, and humidity effect
O_2_	334 MHz Langasite reflective DL [135]	Sputtered ZnO	LL = 20%	O_2_ in N_2_, @ 500 °C to 700 °C
CO	07.2 MHz ZnO/64° Y-X LiNbO_3_ 2-port Res [104]	Drop-casted polyaniline/In_2_O_3_ composite	LL = 60 ppm	CO, H_2_, NO_2_ in synthetic air, @RT
Aromatic and polar compounds	700 MHz AlN DL [162]	Imprinted polymers (for aromatic) and polyeurethane (for polar), spin coating	LL as low as 0.5 ppm for some vapors.	Benzene, toluene and xylene, ethanol, butane, and propane in air, @25–35 °C temperature effect
VOCs	433 MHz dual-port Res (commercial) [78]	Electro-sprayed ZnO	PCA analysis, concentration range:100–5000 ppm	Acetone, trichloroethylene, chloroform, ethanol, propanol, methanol in air @22 °C, humidity effect
Organophosphorus compounds	434 MHz Y-Z LiNbO_3_ reflective DL, [57]	SXFA, solvent-evaporation	η ~ 20°/mgm^−3^, LL < 0.5 mg/m^3^	DMMP in N_2_, @25 °C, 85 °C, wireless sensor
Explosives and CWAs	36–434 MHz Quartz, LiNbO_3_ and ZnO/glass Res or DL [66]	Drop-dried polymers	η as low as 8.3 Hz/ppb, LL as low as 3 ppb	TNT, DNT, Sarin, and DMMP in N_2_, @RT
